# Caesarean section rates and indications: a cross-sectional study from a tertiary care centre in Türkiye

**DOI:** 10.1186/s12884-025-08494-z

**Published:** 2025-11-24

**Authors:** Gazi Guner, Can Berk Karabudak

**Affiliations:** 1 Department of Gynaecologic Oncology, Basaksehir Cam and Sakura City Training and Research Hospital, Istanbul, Turkiye Turkey; 2 Department of Obstetrics and Gynaecology, Basaksehir Cam and Sakura City Training and Research Hospital, Istanbul, Turkiye Turkey

**Keywords:** Caesarean section, Previous uterine surgery, Foetal distress, Turkiye

## Abstract

**Background:**

Globally, including in Türkiye, caesarean section (C-section) rates have been rising steadily, largely due to changes in clinical practices, maternal preferences, medico-legal concerns, and healthcare system dynamics. This study aims to investigate the impact of a high patient load on C-section rates and their indications within a tertiary care setting.

**Methods:**

This retrospective study was conducted on women with deliveries over four years, from June 1, 2020, to May 1, 2025. For pairwise comparisons, numerical and nominal variables were analysed with independent t-test or Pearson’s chi-squared test, respectively. A p-value < 0.05 was accepted as statistically significant. All variables were expressed with 95% confidence intervals (CI).

**Results:**

A total of 68,944 deliveries were eligible for analysis, with an overall C-section rate of 55.0%. Among the indications C-section, the most common was previous uterine surgery, including prior C-section and/or myomectomy, accounting for 56.2% of cases. This was followed by foetal distress (24.3%). Other foetal-related indications included multiple gestation (6.2%), foetal malpresentation (4.2%), and macrosomia (1.9%).Compared with women who had a normal vaginal delivery (NVD), those undergoing C-section had significantly higher percentage of male neonates, maternal age >35 years, multiparity, all categories of preterm birth, early-term birth, low birth weight, and macrosomia.

**Conclusion:**

Our findings indicate that the overall C-section rate was 55.0%. Among the indications for C-section, the most common was previous uterine surgery, which includes prior C-section and/or myomectomy, followed by foetal distress.

## Introduction

Caesarean section (C-section) is a surgical procedure involving incisions through the abdominal wall (laparotomy) and uterus (hysterotomy) to deliver a baby. It is typically indicated when vaginal delivery poses greater risks to the mother or foetus, such as in cases of labour complications, obstructed labour, abnormal foetal presentation, placental abnormalities, or a history of previous C-section. Although C-section is often life-saving in high-risk situations, it remains a major surgical intervention associated with potential complications, including infection, haemorrhage, thromboembolic events, and longer maternal recovery times. Furthermore, C-section may have implications for future pregnancies, increasing the risk of conditions such as placenta accreta spectrum and uterine rupture. Despite these concerns, C-section remains a cornerstone of obstetric care [1]. Globally, including in Türkiye, CS rates have been rising steadily, largely due to changes in clinical practices, maternal preferences, medico-legal concerns, and healthcare system dynamics. While C-section can be a life-saving intervention in the management of pregnancy-related complications, its excessive and non-medically indicated use has raised significant public health concerns [2].The World Health Organization (WHO) has long provided guidance on optimal C-section use. In its 2021 update, the WHO emphasized that CS rates of up to 10% are associated with significant reductions in maternal and neonatal mortality. However, rates above this threshold do not appear to confer additional mortality benefits and may indicate unnecessary surgical intervention [3, 4]. Importantly, WHO does not endorse a specific target rate for all countries, recognizing that appropriate C-section rates may vary depending on population needs and healthcare access.

Basaksehir Cam and Sakura City Hospital, established in 2020, is currently the second-largest tertiary healthcare centre in Türkiye. As a high-volume referral institution, it plays a critical role in managing complex obstetric cases. This study aims to investigate the impact of a high patient load on C-section rates and their indications within a tertiary care setting. Particularly, it seeks to document the C-section rates and their clinical indications in this large-scale referral centre, contributing to a better understanding of surgical delivery trends in high-capacity hospitals.

## Materials and methods

### Study design and participants

A total of 68,944 patients had deliveries at the Department of Obstetrics of Basaksehir Cam and Sakura City Training and Research Hospital over four years, from June 1 2020 to May 1, 2025, of which 37,923 underwent C-section. This study included these 68,944 patients, and data were reviewed retrospectively.

This retrospective study was conducted on women with deliveries, over four years, from May 1, 2025 to from June 1, 2020.

### Data collection

Data on deliveries were retrieved from the Electronic patient file system, which included the mode of delivery, C-section indications and patients’ obstetric characteristics. These characteristics included maternal age, parity, number of parities, number of foetuses, gestational age (GA) at delivery, stillbirths, APGAR score, and birth weight (BW). The inclusion criteria were all pregnancies with more than 20 weeks of gestation, single and multiple pregnancies. Exclusion criteria were less than 20 weeks of gestation.

### Definitions

Maternal anaemia and low birth weight (LBW) were defined according to the WHO criteria; that is, a blood haemoglobin level of less than 11 g/dL and a birth weight of less than 2,500 g (g), respectively [5, 6]. A birth weight of more than 4,000 g was considered macrosomia. Preterm birth is defined as the birth of a baby before 37 weeks of pregnancy. Based on gestational age, preterm births are classified into the following sub-categories: extremely preterm, very preterm, and moderate to late preterm are less than 28 weeks, 28 to less than 32 weeks, and 32 to less than 37 weeks, respectively [7]. Term birth is defined as the birth of a baby at 37 completed weeks of gestation or later. Term births are further categorized based on gestational age: early term, full term, late-term, and post-term are 37 0/7 weeks through 38 6/7 weeks, 39 0/7 weeks through 40 6/7 weeks, 41 0/7 weeks through 41 6/7 weeks, and 42 0/7 weeks and beyond, respectively [8].

### Ethics approval and consent to participate

The study protocol was approved by the Basaksehir Cam and Sakura City Training and Research Hospital Institutional Review Board (IRB)(Permission date and number: 29/01/2025 and E-96317027-514.10.10.10.10.10.10.10.10.10.10–267775454). Obtaining informed consent was waived by the Basaksehir Cam and Sakura City Training and Research Hospital IRB because of the study’s retrospective design. All the procedures were conducted in compliance with the Declaration of Helsinki. Analysis and reporting of the results are in compliance with the Strengthening the Reporting of Observational Studies in Epidemiology checklist.In Turkiye, the legal age for a woman to become pregnant is 18 years old. However, for women who are 17, parental consent may be required under certain circumstances. Individuals aged 16 to 17 are generally not permitted to become pregnant without a judge’s approval. In this study, all Turkish participants were older than 16. Those younger than 16 were all refugees who were not subject to Turkish legislation.

### Data processing and analysis

Data were analysed using Statistical Package for Social Sciences (Armonk, NY, United States of America: International Business Machines Corp.) (SPSS) 29.02. Quantitative data were expressed as means, standard deviation (SD), median, minimum, and maximum, and qualitative data as frequencies and percentages. The Shapiro-Wilk normality test was used to check whether continuous variables were normally distributed For pairwise comparisons, numerical and nominal variables were analysed with Independent T-test and Pearson’s chi-squared test, respectively. Regression analysis was performed to determine the between C-section and maternal-fetal parameters. A p-value of less than 0.05 was accepted as statistically significant. All variables were expressed with 95% confidence intervals (CI).

## Results

### Sociodemographic and obstetric characteristics

During this period, a total of 68,944 women were admitted for delivery and included in the analysis. The mean age of the participants was 28.0 ± 5.8 years. Of the total cohort, 56,166 women (81.5%) were of Turkish nationality, while 12,778 (18.5%) were identified as refugees. The overall mean gestational age at delivery was 37.7 ± 2.9weeks, with a range of 22 to 42 weeks. Preterm and term delivery accounted for 19.6% and 80.4%, respectively. The overall C-section rate was 55%, with 21.1 for primary and 30.9% for previous C-section. The overall CS rate was 55%, with primary C-section accounting for 24.1% and previous C-section accounting for 30.9%. Turkish cases and refugee individuals were rates of C-section at 56.2% and 49.6%, respectively. The mean birth weight of the newborns was 3,040 ± 684 g, with low birth weight (< 2,500 g) observed in 15.1% of cases and macrosomia (> 4,000 g) in 4.5%. The clinical, obstetric, and sociodemographic characteristics of the patients are summarized in Table 1.

**Table 1:** Sociodemographic, clinical and obstetrics characteristics of the participants

Characteristics	Number (*n* = 68,944)	Percentage (%)
Age (years) (mean ± SD)	28.0 ± 5.8	
< 18	593	0.9
≥ 18 < 35	58,071	84.2
≥ 35	10,280	14.9
Nationality		
Turkish	56,166	81,5
Refugees	12,778	18,5
Parity		
Primiparity	24,429	35.4
Multiparity	44,515	64.6
Gestational age at delivery, weeks	37.7 ± 2.9	
Preterm	13,506	19.6
Extremely preterm	1339	1.9
Very preterm	1550	2.2
Moderate to late preterm	10,617	15.4
Term	55,438	80.4
Early term	24,185	35.1
Full term	25,757	37.4
Late term	5228	7.6
post term	268	0.4
Mode of delivery		
NV	31,021	45.0
C-section	37,923	55.0
C-section	37,923	
Primer C-section	16,582	24.1
Previous C-section	21,341	30.9
Foetal outcomes^b^ APGAR 1.minute (mean ± SD) APGAR 5.minute (mean ± SD)	7.9 ± 0.9 9.3 ± 0.6	
Live birth	67,837	98.4
Stillbirth	1,107	1.6
Birth weight (g)^a^	3040 ± 684	
<1,000	1438	2.1
≥ 1,000 < 1,500	1241	1.8
≥ 1,500 < 2,500	7746	11.2
≥ 2,500 < 4,000	55,409	80.4
≥ 4,000	3110	4.5
Sex		
Male	35,033	50,8
Female	33,911	49.2

*N* Number, *% *Percentage, *SD* Standard deviation, *CS* Caesarean section, *g G*ram

^a^Rate calculated per total number of babies born

^b^foetal outcomes include live births and stillbirths

The results of between-group comparisons, including C-section and NVD, are summarised in Table 2. Mothers who underwent C-section had significantly higher percentage of the following factors compared to those who NVD: male gender (51.5% vs. 48.5%, *p* < 0.001), age more than 35 years (18.9% vs. 10%, *p* < 0.001), multiparity (67.1% vs. 61.5%, *p* < 0.001), all preterm births sub-categories (extremely preterm, very preterm, and moderate to late preterm), early term, birth weight < 1000 2.3% vs. 1.8%, *p* < 0.001), 1000–1499 (2.8% vs. 0.6, *p* < 0.001), 1500–2499 (15.4% vs. 61.%, *p* < 0.001), and > 4000 (5.4% vs. 3.4%, *p* < 0.001), but significantly lower stillbirth (1.1% vs. 2.2%, *p* < 0.001), maternal age < 18 (0.4% vs. 1.4%, *p* < 0.001) and 18–35 years (80.6% vs. 88.6%, *p* < 0.001), full-late and posterm weeks, lower mean birth weight (3080 ± 744 vs. 3153 ± 588, *p* < 0.001), lower mean birth weight (37.2 ± 2.9 vs. 38.4 ± 2.7, *p* < 0.001).

**Table 2:** Comparison of demographic and clinical characteristics between normal vaginal deliveries and caesarean section

Parameter	NVD *n* = 31,021 (%)	C-section *n* = 37,923 (%)	*P*
Nationality			*p < 0.001
– Turkish	24,586 (79.3)	31,580 (83.3)	
– Refugee	6,435 (20.7)	6,343 (16.7)	
Sex			*p < 0.001
– Female	15,523 (50.0)	18,388 (48.5)	
– Male	15,498 (50.0)	19,535 (51.5)	
Stillbirth			*p < 0.001
– Alive	30,342 (97.8)	37,495 (98.9)	
– Stillbirth	679 (2.2)	428 (1.1)	
Maternal Age (years) mean ± SD	26.7 ± 5.5	29.1 ± 5.8	*p < 0.001
Age Groups			*p < 0.001
– <18 years	436 (1.4)	157 (0.4)	
– 18–35 years	27,491 (88.6)	30,580 (80.6)	
– >35 years	3,094 (10.0)	7,186 (18.9)	
Parity			*p < 0.001
– Primiparous women	11,953 (38.5)	12,476 (32.9)	
– Multiparous women	19,068 (61.5)	25,447 (67.1)	
APGAR score mean ± SD			
–1.minute	7.7 ± 1.2	7.9 ± 1.2	NS
–5.minute	9.3 ± 0.9	9.4 ± 0.9	NS
Birth Weight (g) mean ± SD	3,153 ± 588	3,080 ± 744	*p < 0.001
Birth Weight Groups			*p < 0.001
– <1000	555 (1.8)	883 (2.3)	
– 1000–1499	175 (0.6)	1,066 (2.8)	
– 1500–2499	1,904 (6.1)	5,842 (15.4)	
– 2500–3999	27,336 (88.1)	28,073 (74.0)	
– >4000	1,051 (3.4)	2,059 (5.4)	
Gestational Age (weeks) mean ± SD	38.4 ± 2.7	37.2 ± 2.9	**p < 0.001
Gestational Age Groups			*p < 0.001
– Extremely preterm	560 (1.8)	779 (2.1)	
– Very preterm	286 (0.9)	1,264 (3.3)	
– Moderate to late preterm	2,749 (8.9)	7,868 (20.7)	
– Early term	8,444 (27.2)	15,741 (41.5)	
– Full term	15,613 (50.3)	10,144 (26.7)	
– Late term	3,210 (10.3)	2,018 (5.3)	
– Post-term	159 (0.5)	109 (0.3)	

*NVD* Normal Vaginal Delivery, *C-section *Cesarean Section

Statistical significance assessed using *chi-square test or *independent t-test as appropriate, NS Non-significant)

Bolded p-values indicate statistically significant differences (p < 0.05)

The specific indications for C-section, along with the number of patients and percentage distributions, are detailed in Table 3. Among the indications for C-section, the most common was previous uterine surgery (including previous C-section and/or myomectomy), accounting for 56.2% of cases. This was followed by foetal distress (24.3%). Multiple gestation (6.2%), foetal malpresentation (4.2%), and macrosomia (1.9%) were also common foetal contributors.

**Table 3:** Distribution of maternal, foetal, and combined maternal-foetal indications for C-section, including number of cases and percentage of total. HIV: human immunodeficiency virus, HSV: herpes simplex virus, CS: Cesarean section

Parameters	*N* = 37,923 (%)
Maternal	22,332 (58.9)
Previous C-section or uterine surgery	21,295 (56.2)
Maternal comorbidities (e.g., cardiac disease, pulmonary disease, chronic or gestational hypertension, he)	643 (1.7)
Preeclampsia or eclampsia	358 (0.9)
Maternal vertically transmitted infection (HIV, HCV, HSV, etc.)	30 (0.1)
Maternal request	6 (0.0)
Foetal	14,008 (36.9)
Foetal distress	9234 (24.3)
Multiple gestation	2357 (6.2)
Foetal malpresentation	1595 (4.2)
Foetal macrosomia	738 (1.9)
Cord prolapses	62 (0.2)
Foetal gross congenital anomalies (teratoma, hydrocephaly, severe cardiac disease, etc.)	22 (0.1)
Maternal-foetal	1583 (4.1)
Labour dystocia	799 (2.1)
Placental invasion anomalies/antepartum haemorrhage (e.g., PAS, placenta previa, vasa previa, placenta abruption)	425 (1.1)
Cephalopelvic disproportion	359 (0.9)

*HIV* Human immunodeficiency virus, *HSV* Herpes simplex virus, *CS* Cesarean Section

### Regression analysis

Regression analysis The results of the regression analysis are summarised in Table 4 and Table 5. Univariate logistic regression analysis identified several factors associated with an increased likelihood of C-section compared with NVD. Increasing maternal age, very low birthweight, low birthweight, macrosomia, male fetal sex, livebirths, maternal age > 35 years, full-term birth, extremely preterm birth, very preterm birth, moderate-to-late preterm birth, early-term birth, and multiparity were each associated with higher odds of C-section.

**Table 4:** Binary univariate logistic regression of maternal and Fetal–Neonatal factors associated with C-section

Category	Variables	β	SE	Z	*p*	OR	95% CI
Maternal	Age (years)	0.0771	0.00142	54.4	*p* < 0.001	1.1	1.0–1.1
	Parity	0.2458	0.0160	15.37	*p* < 0.001	1.3	1.2–1.3
	Birthweight (g)	–3.98 × 10⁻⁴	1.19 × 10⁻⁵	–33.5	*p* < 0.001	1.0	1.0–1.1
	Turkish-refugees	0.2647	0.0196	13.49	*p* < 0.001	1.3	1.3–1.4
	Maternal age groups (ref: 18–35 y)						
	<18 years	–1.128	0.0935	–12.1	*p* < 0.001	0.3	0.3–0.4
	>35 years	0.736	0.0231	31.9	*p* < 0.001	2.1	2.0–2.2
Fetal/Neonatal	Male (vs. female) sex	0.0621	0.0153	4.06	*p* < 0.001	1.1	1.0–1.1
	Alive (vs. stillbirth)	0.673	0.0622	10.82	*p* < 0.001	2.0	1.7–2.2
	Birthweight (g)	−3.98 × 10⁻⁴	1.19 × 10⁻⁵	–33.5	*p* < 0.001	1.000	1.0–1.1
	Birthweight categories (ref: 2500–3999 g)						
	<1000 g	0.4378	0.0548	7.98	*p* < 0.001	1.55	1.4–1.7
	1000–1499 g	1.7803	0.0820	21.71	*p* < 0.001	5.93	5.1–7.0
	1500–2499 g	1.0945	0.0277	39.48	*p* < 0.001	2.99	2.8–3.2
	>4000 g	0.6459	0.0389	16.62	*p* < 0.001	1.91	1.8–2.1
	Gestational age (weeks)	–0.180	0.00350	–51.6	*p* < 0.001	0.835	0.8–0.9
	Gestational categories (ref: Full term)						
	Extremely preterm	0.7613	0.0569	13.39	*p* < 0.001	2.1	1.9–2.4
	Very preterm	1.9173	0.0667	28.74	*p* < 0.001	6.8	6.0–7.8
	Moderate–late preterm	1.4828	0.0256	58.00	*p* < 0.001	4.41	4.2–4.6
	Early term	1.0540	0.0186	56.78	*p* < 0.001	2.87	2.8–3.0
	Late term	–0.0329	0.0311	–1.06	*p* = 0.290	0.97	0.9–1.0
	Post-term	0.0537	0.1250	0.43	*p* = 0.668	1.06	0.8–1.3

*SE* Standard estimate, *OR* Odds Ratio, *Ref* Reference

**Table 5:** Binary multivariate logistic regression of maternal and Fetal–Neonatal factors associated with C-section

Category	Variables	Estimate	SE	Z	*P*	AOR	95% CI
Maternal	Maternal age	0.0801	0.0021	37.43	*p* < 0.001	1.1	1.0–1.1
	Maternal age group (ref: 18–35 years)						
	<18 years	–0.5000	0.1013	–4.94	*p* < 0.001	0.6	0.5–0.7
	>35 years	–0.2937	0.0341	–8.62	*p* < 0.001	0.745	0.7–0.8
	Multiparous vs. primiparous	0.2879	0.0179	16.08	*p* < 0.001	1.334	1.3–1.4
	Nationality Turkish-Refugees	0.1287	0.0216	5.96	*p* < 0.001	1.137	1.1–1.2
Fetal/Neonatal	Infant sex (male vs. female)	0.0369	0.0168	2.20	*p* = 0.028	1.038	1.0–1.1
	Alive vs. stillbirth	1.8934	0.0780	24.29	*p* < 0.001	6.642	5.7–7.7
	Birthweight	4.92 × 10⁻⁵	2.69 × 10⁻⁵	1.83	*p* = 0.067	1.000	1.0–1.1
	Birthweight categories (ref: 2500–3999 g)						
	<1000 g	0.9996	0.1585	6.31	*p* < 0.001	2.717	2.0–3.7
	1000–1499 g	1.5542	0.1257	12.36	*p* < 0.001	4.731	3.7–6.1
	1500–2499 g	0.5353	0.0405	13.21	*p* < 0.001	1.708	1.6–1.9
	>4000 g	0.8564	0.0477	17.95	*p* < 0.001	2.355	2.2–2.6
	Gestational week at birth	–0.1732	0.0139	–12.48	*p* < 0.001	0.841	0.8–0.9
	Gestational age categories (ref: Full term)						
	Extremely preterm	–2.0259	0.2244	–9.03	*p* < 0.001	0.132	0.1–0.2
	Very preterm	–0.2354	0.1468	–1.60	*p* = 0.109	0.790	0.6–1.1
	Moderate–late preterm	0.5866	0.0624	9.40	*p* < 0.001	1.798	1.6–2.0
	Early term	0.7314	0.0299	24.47	*p* < 0.001	2.1	2.0–2.2
	Late term	0.3304	0.0394	8.39	*p* < 0.001	1.4	1.3–1.5
	Post-term	0.5797	0.1352	4.29	*p* < 0.001	1.8	1.4–2.3

*SE* Standard estimate, *AOR* Adjusted Odds Ratio, *Ref* Reference

In the multivariable logistic regression model, increasing maternal age, very low birthweight, low birthweight, macrosomia, male fetal sex, livebirths, maternal age > 35 years, full-term birth, extremely preterm birth, moderate-to-late preterm birth, early-term birth, late-term birth, post-term birth, and multiparity remained independently associated with higher odds of CS.

## Discussion

In our study, we evaluated the C-section indications at tertiary care, with C-section rates of a sizable sample size (*n* = 68,944), and an extended enrolment duration (over 4 years). The overall C-section and primary C-section rates in our sample were notably high at 55.0% (56.1% for Turkish women, 49.7% for refugee women) and 24.1%, respectively, which means that a quarter of women in our sample had a caesarean delivery for the first time. In a study involving 593 adolescent women, the rates of C-section among these individuals were found to be 26.5%. Our findings align with national data indicating persistently high CS rates in Türkiye. Our findings underscore the importance of implementing hospital-level auditing systems using the Robson classification to monitor and reduce unnecessary CS procedures.In a large number of studies from Turkiye, overall C-section rates ranged from 51.9 to 57.6%, which was consistent with our finding [9–12]. One of these studies analysed national data over a five-year period, from 2018 to June 2023, involving 6,161,976 women. The overall and primary C-section rates were 57.55%. and 28.83%, respectively [9]. The latter study, over four years, involved 5,323,500 mothers, resulting in a 51.9% overall C-section rate [10]. In the third of these studies, which included 1,189,578 women, the overall CS rate was 54.6%, varying by hospital type, with rates of 38.6% in state hospitals, 68.9% in university hospitals, and 70.6% in private hospitals [11]. The last study examined 1,266,300 women across 1,503 healthcare facilities, including 879 state hospitals, 557 private hospitals, and 67 university hospitals. The overall C-section rate was 51.2%. However, this study also demonstrated significant differences by hospital type: state hospitals had the lowest rate at 39.7%, while private and university hospitals showed higher rates of 70.6% and 70.3%, respectively [12]. These findings highlight a persistent trend of increased CS rate in Turkiye, particularly in private and academic healthcare settings. The significantly higher rates in private hospitals may be influenced by financial incentives, patient preferences, and time management considerations, as elective C-section can offer greater scheduling convenience for both patients and physicians. University hospitals, although expected to follow evidence-based protocols, may experience high rates due to the complexity of cases, referral patterns, medico-legal concerns, and institutional culture that favours surgical delivery.

A systematic review and meta-analysis of European countries reported C-section rates ranging from 16% to 52.2% over a four-year period, with most countries recording CS rates lower than those observed in Türkiye [13]. In contrast, studies from lower-resource settings such as Burkina Faso, Ethiopia, and Somalia reported overall C-section rates of 30.6%, 34.7%, and 38.6%, respectively [14–16]. These figures illustrate a global dichotomy. These data indicate that, while limited access to surgical delivery remains a public health concern in some low-income countries, the more prominent issue in Türkiye is the overutilization of cesarean sections. This pattern highlights the need for policy interventions and clinical strategies aimed at reducing non-medically indicated C-section procedures.

In our study, the most common indication for C-section was previous uterine surgery, including women with a history of prior CS and myomectomies, which accounted for 46% of all C-section cases. Foetal indications represented 36.9% of all cases, with foetal distress being the leading cause, observed in 24.3% of C-section deliveries. Other notable foetal indications included multiple gestations (6.2%), foetal malpresentation (4.2%), and macrosomia (1.9%). Our results are consistent with previous studies conducted in Türkiye. One study identified previous uterine surgery as the most common indication for C-section, followed by foetal distress and cephalopelvic disproportion (CPD) [17]. Similarly, another study reported previous uterine surgery as the leading cause, followed by foetal distress, with other common indications including malpresentation (breech, transverse, oblique, or foot presentation), multiple pregnancies, and CPD [18]. A large-scale Turkish study involving 69,051 cases reported similar patterns, with previous caesarean sections accounting for 46.1% of cases, followed by foetal distress (13.2%) and cephalopelvic disproportion (8%) [19]. Internationally, indication patterns show regional variability. A systematic review and meta-analysis that included 23 studies from Ethiopia identified CPD as the most common indication for C-section, followed by foetal distress [20]. Similarly, In a systematic review and meta-analysis that included 44 studies from Nigeria, previous uterine surgery was reported as the leading cause, followed by hypertensive disorders, CPD, foetal distress, obstructed labour, antepartum haemorrhage, and multiple gestations [21]. In a study from Poland, the most frequent obstetric indication for C-section was prior C-section, whereas psychological disorders were the most common non-obstetric reason [22]. These comparisons highlight both global similarities and regional differences in C-section indications, influenced by factors such as clinical practice guidelines, access to healthcare resources, referral systems, and patient demographics. The predominance of repeat C-section underscores the need for strategies focused on reducing primary C-section rates and promoting vaginal birth after C-section (VBAC) where medically appropriate. Equally important is the standardization of diagnostic criteria, particularly for indications like foetal distress, to prevent unnecessary surgical interventions and optimize maternal and neonatal outcomes.

In a study indicate that The Dermabond™ Prineo™ Skin Closure System (DP; Ethicon, Somerville, NJ) was generally a safe and effective alternative to conventional wound-closure techniques. Reported complications, such as superficial or deep wound issues, infections, delayed healing, and dehiscence, were comparable to those seen with traditional methods. Several studies highlight the advantages of DP, including reduced operative time and cost, improved cosmetic outcomes, and simplified postoperative wound care. However, allergic contact dermatitis remains an important adverse effect, representing a rare but clinically relevant type IV hypersensitivity reaction. Awareness and early recognition of this complication are essential, particularly in patients with a history of adhesive allergies.

Our comparative analysis of women who underwent NVD versus C-section revealed several significant differences. Turkish nationals were more likely to deliver by C-section than refugee women, suggesting variations in healthcare access or decision-making. Among older mothers, particularly those aged ≥ 35 years, multiparous women and male infants were significantly more common among C-section births. This trend may be attributed to the well-documented association between increasing maternal age, previous uterine surgery, and elevated risk of obstetric complications, which can influence the decision to perform a C-section. Additionally, all categories of preterm birth, including extremely preterm, very preterm, and moderate to late preterm, as well as early-term deliveries were significantly more common among C-section births. Conversely, full-term and post-term births were significantly more common in the vaginal delivery group. These patterns suggest that cesarean delivery is more commonly employed in the context of maternal or fetal complications. Conditions such as preeclampsia, intrauterine growth restriction (IUGR), and maternal systemic diseases likely contribute to the increased incidence of early or preterm cesarean interventions. Furthermore, Stillbirths were significantly lower in the C-section group, which may reflect a possible bias toward surgical intervention in high-risk pregnancies.

Birth weight distributions differed significantly between the NVD and C-section groups. Low birth weight infants (< 2500 g) were notably more prevalent in the C-section group, indicating a greater reliance on CS in the management of preterm or growth-restricted pregnancies. In contrast, normal birth weight infants (2500–3999 g) were more commonly delivered vaginally. Notably, macrosomia infants (> 4000 g) were more commonly delivered by C-section, likely due to concerns about shoulder dystocia, labour complications, or cephalopelvic disproportion. These findings highlight the critical influence of foetal weight on clinical decision-making regarding the mode of delivery, with both ends of the birth weight spectrum significantly associated with increased rates of C-section intervention.

Beyond clinical factors, Türkiye’s rising caesarean rates may also reflect medico-legal pressures and convenience-driven practice patterns, which can discourage vaginal birth. Strengthening support for VBAC through clearer guidelines, staff training, and improved facility readiness could help reduce repeat C-section.The lower C-section rates observed among refugee women may indicate cultural preferences or, more concerningly, barriers to accessing intrapartum interventions. Ensuring equitable, culturally sensitive maternity care is essential.Overall, actionable strategies to reduce unnecessary C-section include expanding midwife-led labour support, standardising decision-making processes, promoting VBAC programmes, improving medico-legal protections, and enhancing antenatal counselling to support informed choice [23].

### Strengths and limitations

This study’s primary strength is its large sample size, which provides robust statistical power and increases the generalizability of the results. Although conducted in a single tertiary centre, findings may not fully represent national diversity in healthcare access or patient demographics. A key advantage is the hospital’s adherence to strict protocols for data entry, ensuring that birth reports are thoroughly recorded in the electronic delivery system. Therefore, there is no incomplete or missing data in our records. The main limitation of our study is its retrospective study.

## Conclusion

The overall C-section rate in our cohort was 55.0%. The most common indications for C-section were previous uterine surgery and foetal distress. The findings also reveal a notable association between maternal age, parity, and the likelihood of undergoing a C-section, with older mothers, multiparous women, and those with male infants being more likely to C-section. Preterm deliveries and cases of LBW and macrosomia were more commonly managed with C-section, suggesting that C-section remains the preferred mode of birth for high-risk pregnancies. Underscoring the need for strategies aimed at reducing primary C-section rates, promoting VBAC, and standardizing diagnostic criteria, particularly for conditions like foetal distress.

## Data Availability

All data generated or analysed during this study are included in this article. The datasets used and/or analysed during the current study are available from the corresponding author upon reasonable request. However, restrictions apply to the availability of these data, as they were used under license for this study and are not publicly available. The corresponding author (email: dr.gaziguner01@gmail.com) can be contacted for data requests.

## Abbreviations

C-section: Caesarean section
WHO: The World Health Organization
SD: Standard deviation
IRB: Institutional review board
